# Tapia Syndrome and Severe Pain Induced by Occipital Bone Metastasis of Prostate Cancer

**DOI:** 10.7759/cureus.49327

**Published:** 2023-11-24

**Authors:** Yojiro Ishikawa, Ichiro Seto, Satoshi Teramura, Motohisa Suzuki, Yoshiaki Takagawa, Masanori Machida, Kanako Takayama, Nor Shazrina Sulaiman, Yuntao Dai, Yasuhiro Kikuchi, Masao Murakami

**Affiliations:** 1 Radiology, Tohoku Medical and Pharmaceutical University, Sendai, JPN; 2 Radiation Oncology, Southern Tohoku Proton Therapy Center, Koriyama, JPN; 3 Neurosurgery, Southern Tohoku Research Institute for Neuroscience, Southern Tohoku General Hospital, Koriyama, JPN

**Keywords:** 3d conformal radiation therapy, tapia syndrome, tapia's syndrome, prostate cancer, palliative radiation therapy

## Abstract

Tapia syndrome is characterized by unilateral tongue paralysis, hoarseness, and dysphagia. It is often associated with issues in the lower cranial nerves and is rarely caused by malignant tumors.

A 71-year-old Japanese male with prostate cancer and bone metastases experienced severe headaches, oral discomfort, dysphagia, and hoarseness for a month. Neurological examination revealed left-sided tongue atrophy and left vocal cord paralysis, suggesting problems with cranial nerves IX and XII. CT scans showed bone metastasis in the left occipital bone. Brain MRI showed no brain or meningeal metastasis, but neck MRI revealed a mass near the left hypoglossal canal. His prostate-specific antigen (PSA) level was 53.2 ng/mL. Based on these findings, we diagnosed him with occipital bone metastasis of prostate cancer with Tapia syndrome. We treated him with palliative radiation therapy (RT), delivering 30 Gy in 10 fractions over two weeks. We did not use drug treatment or chemotherapy due to side effects and the patient's preferences. After just one day of RT, his severe headache and oral discomfort significantly improved. By the end of the two-week treatment, his hoarseness had also improved, and he was able to eat. However, tongue atrophy had not improved three months after RT.

We presented a unique case of palliative RT for occipital bone metastasis of prostate cancer with Tapia syndrome. Within two weeks, the patient's headache and dysphagia had greatly improved, although tongue atrophy remained partially unresolved after palliative RT.

## Introduction

A group of diseases characterized by laryngeal paralysis as one of their symptoms, combined with various cranial nerve paralyses, is referred to as "mixed laryngeal paralysis." Examples of known conditions in this category include Collet-Sicard syndrome, Vernet syndrome, Schmidt syndrome, Avellis syndrome, and Villaret syndrome [1-3].

Tapia syndrome was first described in 1904 by Spanish otolaryngologist Antonio Garcia Tapia as a lesion outside the central nervous system causing neurological signs and symptoms [4]. Now recognized as a rare complication of airway manipulation, the chief complaint is characterized by unilateral paralysis of the tongue and vocal cords, with dysphonia, tongue deviation, and dysphagia [5]. It is frequently reported in any surgical procedure requiring general anesthesia and tracheal intubation [6-8]. This study describes the progression of a bone metastatic lesion caused by prostate cancer, which presented with symptoms resembling Tapia syndrome.

## Case presentation

A 66-year-old Japanese male underwent proton beam therapy (PBT) for advanced prostate cancer with lymph node metastases following endocrine therapy (gonadotropin-releasing hormone antagonists and antiandrogen). His medical history included essential hypertension. Approximately five years later after PBT, the patient received a diagnosis of recurrence with increased serum prostate-specific antigen (PSA) values, multiple lymph node metastases, and bone metastases. A month after the recurrence diagnosis, the patient began experiencing severe headaches extending from the back of the left eye to the neck. Concerned about the possibility of brain metastasis, the patient consulted a nearby neurosurgeon. However, brain metastasis was ruled out by brain MRI. Nonetheless, the patient developed hoarseness and dysphagia approximately two weeks after consulting the neurosurgeon, prompting further evaluation. The patient also reported an abnormal sensation in the left half of the tongue in the oral cavity.

**Figure 1:**
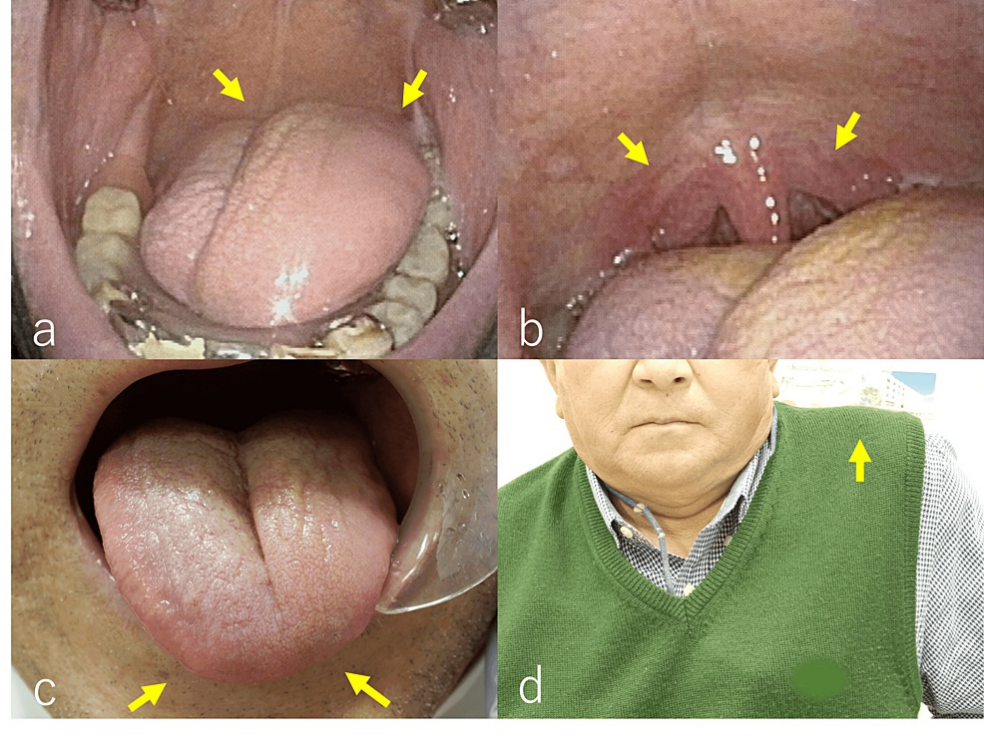
Intraoral gross and examination findings. (a) On the intraoral examination, it was observed that the left side of the tongue appeared to be more enlarged than the right side (yellow arrow). (b) No obvious abnormalities were observed in the soft palate (yellow arrow). (c) On the extraoral examination of the tongue, it was noted that it deviated to the left (yellow arrow). (d) Left shoulder elevation was possible, and no apparent abnormalities of the accessory nerve were observed (yellow arrow).

A physical examination revealed swelling on the left side of the tongue within the oral cavity (Figure 1a), but no abnormalities were observed in the soft palate (Figure 1b). Tongue movement displayed a left-right asymmetry, with the tongue slightly deviating to the left (Figure 1c). There was no noticeable difference in shoulder elevation between the right and left sides (Figure 1d). No other neurologically apparent abnormalities were detected and suggested abnormalities in the XII cranial nerve alone (the hypoglossal nerve).

**Figure 2:**
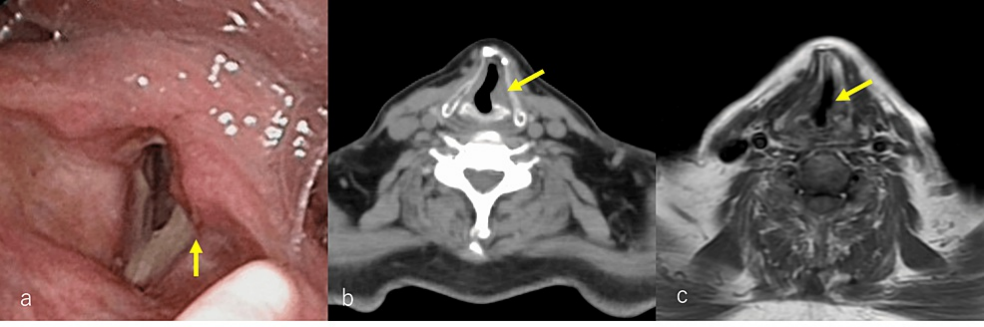
Laryngoscopy, CT, and MRI of the patient. (a) On laryngoscopy, it was observed that the left vocal folds were fixed during inspiration (yellow arrow). (b) CT scan revealed a left-right difference near the vocal folds (yellow arrow). (c) In MRI T1-weighted imaging, a left-right difference near the vocal folds was also indicated (yellow arrow).

**Figure 3:**
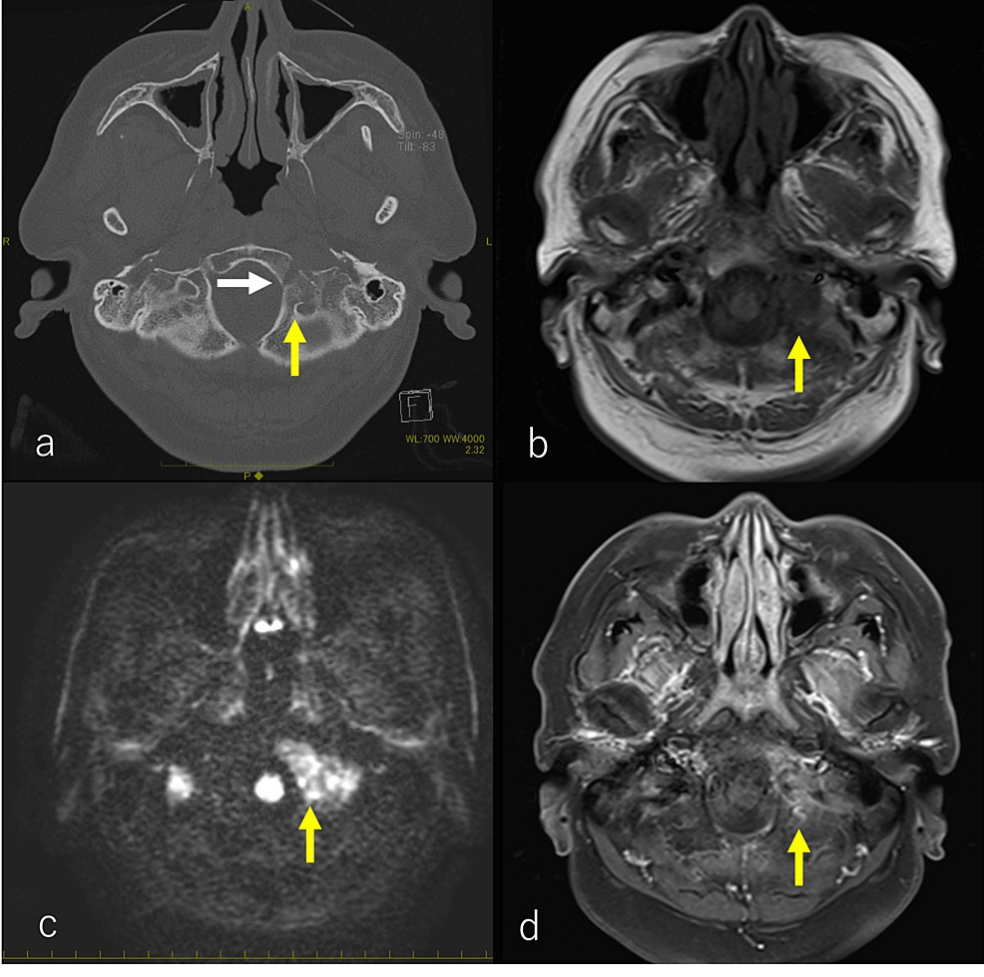
Pretreatment CT and MRI findings. (a) CT bone scan revealed osteolytic bone metastasis extending from the left side of the oblique plateau to the occipital bone (yellow arrow) with invasion into the sublingual neural tube (white arrow). (b) MRI scan showed a low-signal area in the region identified on CT in the T1-weighted image (yellow arrow) and (c) a high signal in the same area in the diffusion-weighted image (yellow arrow). (d) Gadolinium contrast-enhanced CT also demonstrated a contrast enhancement effect (yellow arrow).

**Figure 4:**
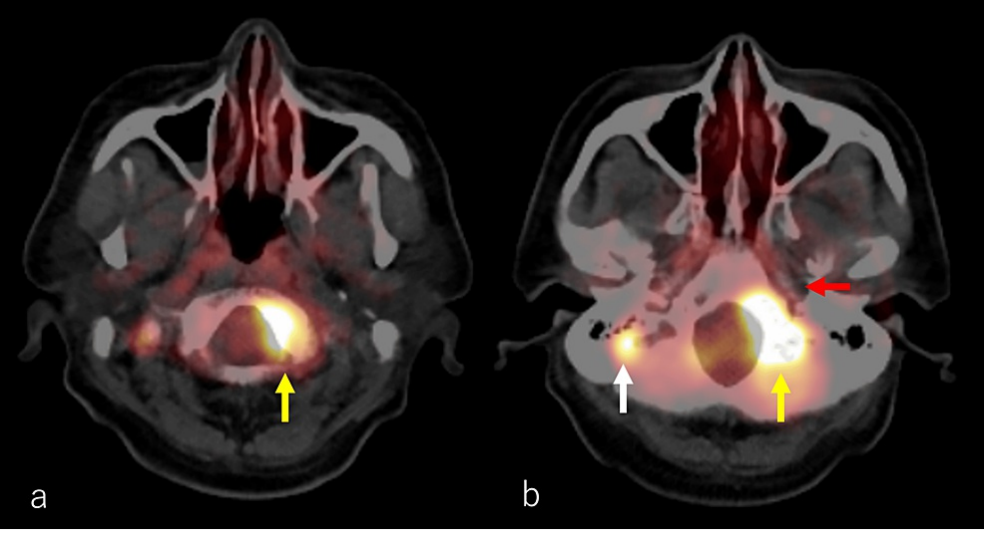
Fluorodeoxyglucose-positron emission tomography. The image showed fluorodeoxyglucose (FDG) uptake extending from the left side of the oblique plateau to the left side of the occipital bone (a, b; yellow arrows). There appeared to be no obvious extension toward the jugular foramen (b; red arrow). A continuous uptake pattern was also observed in the right occipital bone (b; white arrow).

Laryngoscopy, neck CT, and MRI indicated left vocal cord palsy (Figures 2a-2c), while brain CT and MRI revealed an osteolytic lesion extending from the occipital bone to the oblique plateau (Figures 3a-3c). These findings suggest that the symptoms of hoarseness and the observation of vocal fold fixation, as indicated by laryngoscopy, neck CT, and MRI, may be related to the X cranial nerve (the vagus nerve). Additionally, a gadolinium contrast-enhanced MRI scan identified a contrast-enhancing mass (Figure 3d). Fluorodeoxyglucose (FDG)/PET imaging displayed increased metabolic activity within the mass shadow identified on CT and MRI (Figures 4a, 4b). Serum PSA value was 53.2 ng/mL.

Based on the physical and radiological assessments, the diagnosis of occipital bone metastases with Tapia syndrome was confirmed. The patient was scheduled to undergo palliative radiation therapy (RT) to alleviate symptoms.

**Figure 5:**
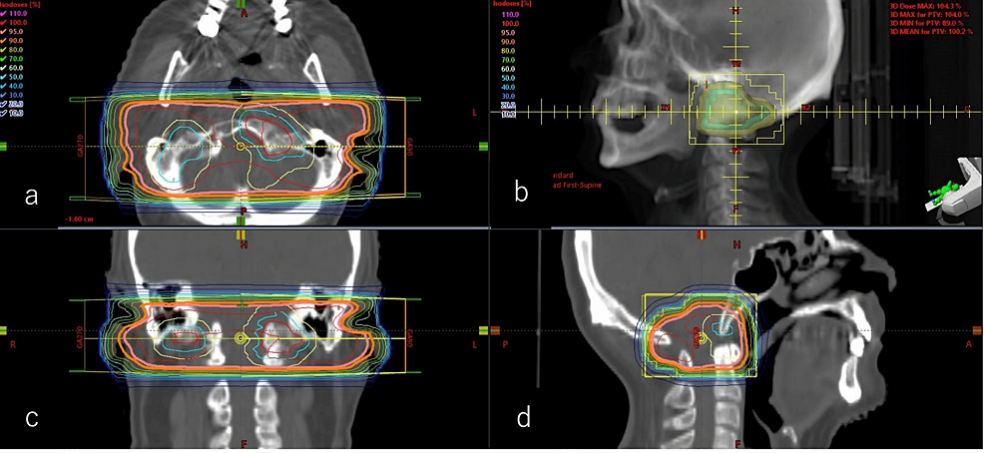
Palliative radiation therapy to alleviate symptoms. Treatment planning was performed using Eclipse (Palo Alto, CA: Varian Medical Systems). Radiotherapy fields are shown in (a) axial image, (b) beam's eye view, (c) coronal image, (d) and sagittal image. The gross tumor volume is outlined by red lines for tumors on both the left and right sides of the occipital bone metastases. The clinical target volume and planning target volume are represented by light blue and yellow lines, respectively. X-ray beams from two directions were performed.

The RT planning was developed using Eclipse (release 6.5; Palo Alto, CA: Varian Medical Systems), designating the osteolytic bone metastasis in the occipital bone as the gross tumor volume, with a CTV margin of 1.0 cm. The prescribed PTV margin was 0.5 cm. The patient received a standard palliative RT dose of 30 Gy delivered in 10 fractions. The 10 MV photon treatments were delivered by a Clinac iX linear accelerator (Palo Alto, CA: Varian Medical Systems) (Figures 5a-5d).

After just one day of palliative RT, the severe headache and oral discomfort significantly improved. One week after RT, the patient experienced improvements in hoarseness. However, objective improvements in terms of tongue displacement and atrophy were not evident. A follow-up MRI scan one month after treatment showed no significant enlargement of the tumor, and the patient reported the disappearance of pain. Tongue atrophy was not completely resolved three months after palliative RT.

## Discussion

In the absence of a history of surgical procedures or direct trauma, it is necessary to suspect an association with lesions, such as left-sided tumors compressing the left recurrent laryngeal nerve, left recurrent laryngeal nerve palsy due to distention of structures in the heart and great vessels, malignant lymph nodes, head and neck tumors, and abscess formation [4]. Previous reports have described schwannomas, lymphomas, and, as in the present case, in patients with prostate cancer. Table 1 summarizes the reported associations between neoplastic lesions and Tapia syndrome as far as we could capture them in a PubMed search of studies in English (available at http://www.ncbi.nlm.nih.gov/pubmed/) [9-12].

**Table 1:** A review of Tapia syndrome related to tumors. M: male; F: female

Studies	Age	Sex	Diagnosis	Treatment	Follow-up	Recovery
Andrioli et al. (1980) [12]	25	M	Neurofibroma	Resection	-	Unknown
Cantalupo et al. (2014) [11]	16	M	Diffuse large B cell lymphoma	Chemotherapy	Three years	Improvement (vocal cord symptoms partially remained)
Yilmaz et al. (2015) [10]	61	M	Prostate cancer	Radiation therapy	-	Unknown
Sánchez-Soblechero et al. (2020) [9]	80	F	Jugular paraganglioma	Best supportive care	-	Unknown
Present case	71	M	Prostate cancer	Radiation therapy	Two months	Improvement (vocal cord symptoms, tongue atrophy partially remained)

Tumor-related Tapia syndrome is treated with chemotherapy, radiation therapy, or surgery as a treatment for the underlying disease [9-12]. This patient had tumor-associated Tapia syndrome, which resulted in very severe pain in addition to the recurrent and hypoglossal nerve palsies characteristic of Tapia syndrome. In past reports, there are few references to pain [9-12]. This case is a clinical presentation of Tapia syndrome with pain symptoms, which is a novelty compared to other reports.

Many of the symptoms of this disease are relatively persistent, and reports examining postoperative complications indicate a recovery time ranging from 3 to 22 months, with a median of 9-12 months [13,14]. Complete recovery occurs in approximately 30% of patients, incomplete recovery in 39% of patients, and no recovery in more than 26% of patients [13,14]. In addition, rehabilitation is considered the mainstay of treatment, and the establishment of a systematic swallowing rehabilitation program, incorporating speech therapy from an early stage, is crucial to achieving a favorable outcome. The role of steroids in the treatment of Tapia syndrome remains controversial, with no clear evidence, but treatment in oral or intravenous form may be used [4,15].

The cure rate for tumor-related Tapia syndrome is unknown. In a similar case of bone metastasis from prostate cancer, the course of symptoms was unknown [10]. In this case, we reported that subjective symptoms disappeared relatively quickly. However, there was no noticeable improvement in symptoms of hoarseness or sublingual nerve palsy.

The treatment considerations in this case suggest that it may be difficult to completely reverse the symptoms of Tapia syndrome itself, although the symptomatic pain is a response similar to that of normal bone metastases [16]. However, the patient in the present study was treated with palliative irradiation, and other results may be obtained if local irradiation is intensified, as in the case of oligometastasis [17]. Another limitation of this case was the limited treatment options available, as the patient experienced severe side effects from drug therapy, rendering drug treatment including chemotherapy challenging.

## Conclusions

We reported an unusual case of palliative RT for occipital bone metastasis of prostate cancer accompanied by Tapia syndrome. Tapia syndrome typically arises from nerve compression during surgical procedures and is rarely caused by tumors. The treatment course of this syndrome is expected to offer valuable insights for oncologists encountering similar cases. In the present study, headache and dysphagia showed marked improvement with RT, nevertheless, tongue atrophy was not completely resolved. There has been no previous report of a clinical course in which cancer pain was added to Tapia syndrome. While this is a rare disease, an early and accurate diagnosis may lead to symptom improvement in patients.
